# Multitarget inhibition of CDK2, EGFR, and tubulin by phenylindole derivatives: Insights from 3D-QSAR, molecular docking, and dynamics for cancer therapy

**DOI:** 10.1371/journal.pone.0326245

**Published:** 2025-06-17

**Authors:** Khadijah M. Al-Zaydi, Soukayna Baammi, Mohamed Moussaoui

**Affiliations:** 1 Department of Chemistry, College of Science, University of Jeddah, Jeddah, Saudi Arabia; 2 Bioinformatics Laboratory, College of Computing, Mohammed VI Polytechnic University, Ben Guerir, Morocco; 3 Laboratory of Physical Chemistry of Applied Materials, Faculty of Sciences Ben M’Sick, Hassan II University of Casablanca, Morocco; University of Alabama at Birmingham, UNITED STATES OF AMERICA

## Abstract

Cancer remains one of the leading causes of death globally, presenting significant challenges to healthcare systems due to its complexity and the limitations of current therapeutic strategies. Despite advancements in anticancer drug development, monotherapies often fail to provide long-term efficacy due to the emergence of drug resistance. This resistance is primarily due to the activation of compensatory pathways in cancer cells, which allows them to bypass the effects of single-target therapies. To overcome this, targeting multiple key proteins simultaneously has emerged as a promising strategy to enhance therapeutic outcomes and address resistance mechanisms. In this study, 2-Phenylindole derivatives were explored as MCF7 breast cancer cell line inhibitors using 3D-QSAR modeling to design more effective compounds. The CoMSIA/ SEHDA model demonstrated high reliability (R² = 0.967) and a strong Leave-One-Out cross-validation coefficient (Q² = 0.814), further validated by external testing (R²_Pred_ = 0.722). Six new compounds with potent inhibitory activity were designed, and their favorable ADMET profiles were confirmed. Molecular docking studies revealed that the newly designed compounds exhibited better binding affinities (−7.2 to −9.8 kcal/mol) to key cancer-related targets (CDK2, EGFR, and Tubulin) compared to the reference drug and the most active molecule (molecule 39) in the dataset. Additionally, 100 ns molecular dynamics simulations confirmed the stability of the best-docked complexes, highlighting their potential as promising candidates for anticancer drug development.

## 1. Introduction

Cancer remains one of the leading causes of death worldwide, posing a significant challenge to healthcare systems due to its complexity and the limitations of current therapeutic strategies [1]. This challenge is further compounded by the fact that, despite advancements in anticancer drug development, monotherapy often fails to achieve long-term efficacy due to the emergence of drug resistance [2]. This resistance arises because a major limitation of single-target therapies is their susceptibility to compensatory pathway activation, which allows cancer cells to bypass drug effects and reduce treatment effectiveness [3]. To address these challenges, targeting multiple key proteins simultaneously has emerged as a promising approach to enhance therapeutic outcomes and overcome resistance mechanisms [4]. Among the most critical molecular targets in cancer therapy are Cyclin-Dependent Kinase 2 (CDK2), Epidermal Growth Factor Receptor (EGFR), and Tubulin, each of which plays a pivotal role in tumor progression and development of drug resistance.

CDK2, a key cell cycle regulator, controls the transition from the G1 to the S phase. Its overactivation leads to unchecked cell division, facilitating rapid tumor growth and contributing to tumor aggressiveness and resistance to apoptosis [5]. EGFR, a receptor tyrosine kinase, is frequently overexpressed or mutated in cancers, activating downstream signaling pathways that promote uncontrolled proliferation, survival, and migration. This dysregulation drives tumor growth, angiogenesis, and metastasis [6]. Tubulin, a structural component of microtubules, is essential for cell division and mitosis. Disruptions in tubulin dynamics can cause chromosomal instability, a hallmark of cancer, and contribute to resistance against microtubule-targeting agents [7]. However, resistance to inhibitors of these proteins often arises due to the development of mutation or activation of alternative survival pathways.

By simultaneously targeting CDK2, EGFR, and Tubulin, this multi-targeted therapy addresses multiple pathways involved in cancer cell survival, proliferation, and metastasis. This approach can potentially prevent or overcome resistance mechanisms that develop with single-target therapies. Moreover, it may enhance treatment efficacy by more effectively controlling tumor growth, reducing the risk of recurrence, and improving patient outcomes. Combining these targets could lead to more durable and potent treatment regimens, particularly for patients with resistant cancers.

The indole nucleus has emerged as a highly versatile scaffold in developing compounds with promising antiproliferative activity, particularly in cancer treatment [8]. A range of 2-phenylindoles has been identified for their ability to inhibit the growth of human breast cancer cells, with the specific mechanisms of action varying depending on the type and position of the substituents on the phenyl ring [9,10]. Notably, recent studies on 2-phenylindole-3-carboxaldehydes have demonstrated their potent antimitotic activity by inhibiting tubulin polymerization, which is crucial for cell division [11]. To address the in-vivo instability of the aldehyde functional group, several modifications, such as the formation of oximes, hydrazones, and other derivatives, were introduced, resulting in compounds with improved stability and continued antimitotic activity [12]. Furthermore, using 3D-QSAR and docking studies, structural optimization of these compounds has provided valuable insights into their interaction with tubulin, EGFR [13], CDK2 [14] particularly at the inhibitor binding site. These advances have motivated further exploration of novel anticancer agents based on the 2-phenylindole scaffold, focusing on synthesizing stable derivatives with enhanced potency.

Given this background, the present study explores the potential of 2-Phenylindole derivatives as multitarget inhibitors against CDK2, EGFR, and Tubulin. Through an integrated computational approach, including 3D-QSAR modeling, molecular docking, and molecular dynamics simulations, we aim to identify novel compounds with strong and stable binding affinities to all three targets. The proposed compounds may exert a synergistic effect by simultaneously inhibiting these key proteins, disrupting different pathways involved in tumor progression and resistance.

## 2. Materials and methods

### 2.1. Data set

**Fig 1:**
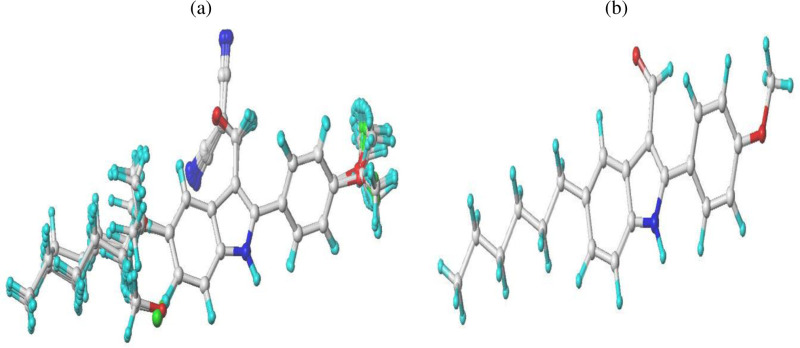
(a) 3D-QSAR structure superposition of training set (b) compound 5n as a template.

A database of thirty-three compounds was compiled from literature sources [12,15], consisting of novel 2-Phenylindole derivatives, which are being investigated as potential anti-breast cancer agents. The dataset was divided into two groups: twenty-eight compounds constituted the training set, while five compounds, randomly selected, formed the test set to evaluate the model’s effectiveness. The chemical structures of the compounds in both the training and test sets are presented in **Fig 1** and **Table 1**. This data was used to develop a 3D-QSAR model and analyze the physicochemical properties of the compounds. For the QSAR analysis, the in vitro biological activity values (IC_50_, in µM) were converted to the corresponding pIC_50_ values (pIC_50_ is the negative logarithm of IC_50_, i.e., pIC_50_ = 6 − log10 (IC_50_)). The 3D structure building and all modeling activities were conducted using the Sybyl 2.0 program package.

**Table 1:** pIC_50_ values of the reported 2-Phenylindole derivatives against EGFR, CDK2 and Tubulin.

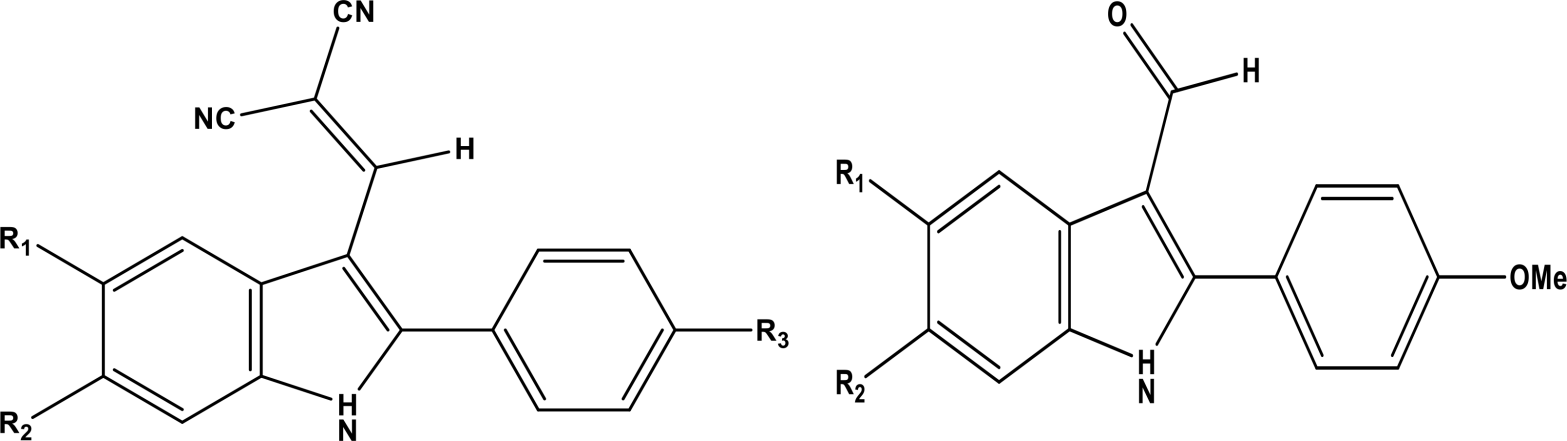								
Compound	R_1_	R_2_	R_3_	pIC_50_	Compound	R_1_	R_2_	pIC_50_
6a	H	H	H	6.721	5a	OMe	H	6.745
6b	H	H	OMe	6.114	5b	H	OMe	6.796
6c	OMe	H	OMe	6.469	5c	H	F	7.367
6d	H	OMe	OMe	6.523	5d	F	H	6.620
6e	F	H	OMe	6.509	5e	H	Cl	7.187
6f	H	F	OMe	6.658	5f	Me	Cl	7.208
6g	H	OMe	Me	6.602	5g	Me	H	6.854
6h	Me	H	OMe	6.699	5h	Pr	H	7.268
6i	Me	Cl	OMe	6.699	5i	*i*-Pr	H	7.013
6j	*n*-Pr	H	OMe	6.721	5j	*n*-Bu	H	7.658
6k	iPr	H	OMe	6.509	5k	*sec*-Bu	H	6.745
6l	*n*-Bu	H	OMe	7.886	5l	*t*-Bu	H	6.237
6m	*n*-Pentyl	H	OMe	7.119	5m	*n*-Pentyl	H	7.699
6n	*n*-Hexyl	H	OMe	7.602	5n	*n*-Hexyl	H	8.222
6o	*n*-Bu	H	Me	7.000				
6p	*n*-Bu	H	Et	6.699				
6q	*n*-Bu	H	CF3	6.824				
6r	*n*-Pentyl	H	CF3	7.000				
6s	*n*-Hexyl	H	CF3	7.167				

### 2.2. Molecular alignment

**Fig 2:**
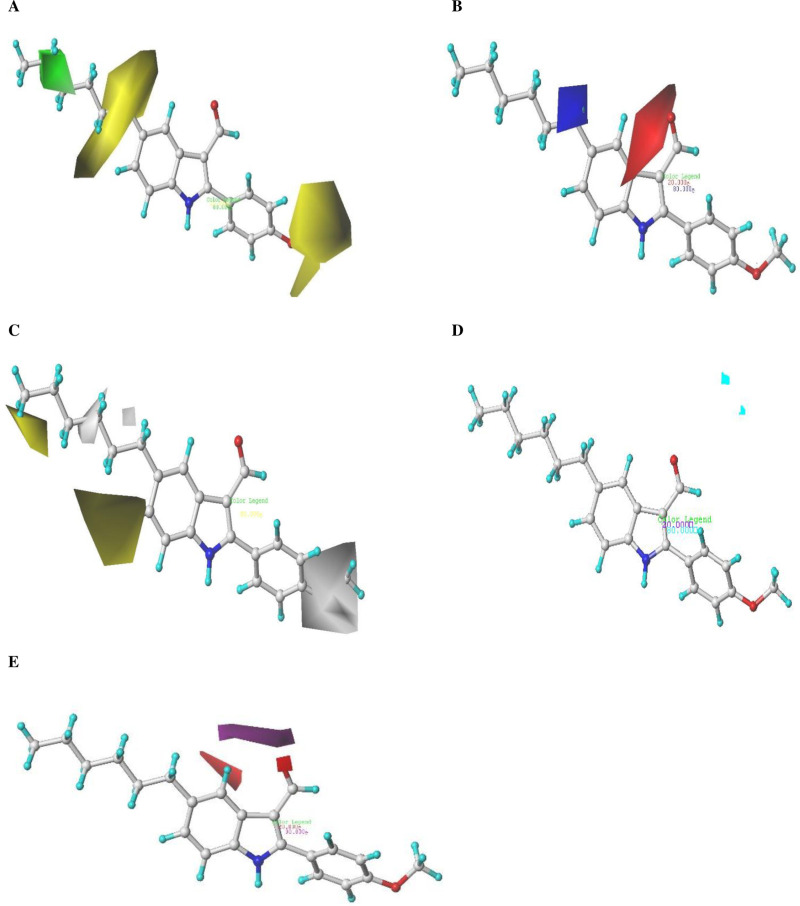
CoMSIA contour maps: (A) Steric, (B) Electrostatic, (C) Hydrophobic, (D) Hydrogen bond donor, and (E) Hydrogen bond acceptor fields, displayed with a grid spacing of 2.0 Å and combined with compound 5n.

Molecular structures were first sketched using the sketch module in the SYBYL program and then optimized with the standard Tripos molecular mechanics force field [16] and Gasteiger-Hückel charges [17], using the conjugate gradient method and a gradient convergence criterion of 0.01 kcal/mol. The next crucial step, molecular alignment, was performed to develop an effective 3D-QSAR model. **Fig 2** presents the 3D structure of the core and the superimposed aligned structures of the dataset. The dataset alignment was done using the distill alignment technique in SYBYL [18], with the most active compound 5n, as the template.

### 2.3. CoMSIA analysis

The descriptor fields of the CoMSIA method were computed within a 3D cubic grid with 2 Å dimensions, extending from the edges of the aligned structures in all directions. At each grid point, the steric, electrostatic, hydrophobic, hydrogen-bond donor, and hydrogen-bond acceptor properties were determined. To quantify these five fields, a probe atom—a charged sp3 hybridized carbon atom with a 1.0 Å radius and a net charge of +1.0—was used at each grid point. The probe atom’s slope parameter, which defines the slope of the Gaussian function, was set to its default value of 0.3 [19].

### 2.4. Partial least squares (PLS) analysis

To evaluate the linear correlation between the CoMFA and CoMSIA descriptors and biological activity values, the PLS method [20] was employed. The leave-one-out (LOO) cross-validation method was used in PLS analysis to determine the optimal number of components (N), based on the highest cross-validation correlation coefficient (Q²) and the lowest standard error of estimation (SEE). After identifying the optimal N, non-cross-validated methods were applied to assess the overall significance of the models, using statistical parameters like the coefficient of determination (R²), the F-value (Fisher test), and the standard error of estimation (SEE). Additionally, several external validation strategies were utilized to further evaluate the robustness and statistical validity of the established models [21]. The equation for SEE is presented below:


$$SEE=\;\sqrt{\frac{PRESS}{n-c-1}}$$

Where n represents the number of compounds, c represents the number of components, and PRESS is the sum of squared deviations between the predicted and actual activity values for each molecule in the test set.

### 2.5. Molecular docking studies

The three-dimensional structures of 3 protein targets, were retrieved from the Protein Data Bank (RCSB) via https://www.rcsb.org/ (**Table 2**). These structures were visualized using UCSF Chimera [22] and prepared with MGLtools (version 1.5.6, The Scripps Research Institute, La Jolla, CA, USA) [22]. Preprocessing involved removing water molecules, heteroatoms (hetatm), and co-crystallized ligands. Subsequently, polar hydrogen atoms were added, Gasteiger charges were assigned, and the structures were converted to pdbqt format for further analysis [23]. The grid box spacing was set to 0.375 Å, centered on the regions where co-crystallized ligands interact with active site residues. Docking simulations were conducted for all 3 protein targets, generating nine poses per protein-ligand complex based on docking affinity. The docking outcomes were visualized and analyzed using Discovery Studio Viewer to identify critical interactions between ligands and protein binding sites [24]. For each protein target, the conformation with the lowest binding affinity (as indicated by docking scores) and the highest number of bonds was chosen as the initial binding mode for subsequent molecular dynamics simulations.

**Table 2:** The selected targets and the coordinates of the grid box.

Protein	PDBID	Grid box center (Å)	Grid box size(Å)
Tubulin	1SA0	center_x = 117.219 center_y = 90.179 center_z = 6.289	size_x = 20 size_y = 18 size_z = 40
Epidermal growth factor Receptor (EGFR)	1M17	center_x = 21.697 center_y = 0.303 center_z = 52.093	size_x = 42 size_y = 18 size_z = 22
Cyclin-dependent kinase (CDK2)	2A4L	center_x = 100.865 center_y = 101.746 center_z = 79.893	size_x = 40 size_y = 40 size_z = 40

### 2.6. Molecular dynamics simulations (MDs)

Molecular dynamics (MD) simulations were performed using GROMACS 2019.3 [25] to evaluate the stability and binding mechanisms of protein-ligand complexes involving the designed and active compounds. The CHARMM27 force field was used to construct protein topologies, while ligand topologies were generated using the SwissParam server [26]. Each complex was positioned within a dodecahedral box (1.0 nm) filled with TIP3P water molecules and neutralized with counter ions [27]. Energy minimization was conducted using the steepest descent algorithm with a maximum force threshold of 1000 kJ/mol/nm [28]. To equilibrate the systems, two consecutive 1 ns simulations were performed under NVT and NPT ensembles at 300 K and 1 bar, regulated by the Berendsen thermostat and Parrinello–Rahman barostat, respectively. Periodic boundary conditions (PBC) were applied throughout the simulations, and long-range electrostatics were calculated using the particle mesh Ewald (PME) method. High-frequency bonds involving hydrogen were constrained using the LINCS algorithm, allowing the use of a 2-fs integration time step [29]. The MD simulations were conducted over 100 ns, totaling 50,000,000 steps, with coordinates recorded every 2 fs. The output trajectories were generated, and the corresponding data files were analyzed to gain a deeper understanding of the protein’s behavior.

## 3. Results and discussion

### 3.1. CoMSIA results

To construct a robust 3D-QSAR model, molecule 5n, which exhibits the highest inhibitory activity, was chosen as a reference for data alignment. This alignment was crucial for generating contour maps in both CoMFA and CoMSIA analyses, as illustrated in **Fig 1**.

The primary objective of this phase is to develop reliable CoMFA and CoMSIA models by correlating the observed and predicted pIC_50_ values for the training and test sets. In the CoMFA model, steric and electrostatic fields were integrated. On the other hand, the CoMSIA model was developed using thirty-one different combinations of steric, electrostatic, hydrophobic, hydrogen-bond donor, and hydrogen-bond acceptor fields. The most effective model was identified by evaluating the highest coefficient of determination values for both non-cross-validation (R²) and cross-validation (Q²), alongside the lowest standard error of estimate (SEE), the minimal number of principal components (N), and the most significant F-test value (F). Among the tested models, the SEHDA CoMSIA model provided the most accurate predictions of biological activity, demonstrating the most favorable statistical metrics **Table 3**. This model achieved an R² of 0.814, utilized six optimal principal components, and had a reliable Standard Error of Estimate (SEE) of 0.091 and an F-test value of 102.992. Additionally, **Table 4** summarizes the predicted pIC_50_ values and CoMSIA/SEHDA descriptors for the compounds analyzed.

**Table 3:** Statistical results of CoMSIA models with different combinations of molecular fields.

Generated model	Q^2^	N	SEE	R^2^	F	R^2^_pred_	Fractions				
**S**	**E**	**H**	**D**	**A**
CoMSIA/S	0.568	2	0.185	0.864	22.325	0.642	1				
CoMSIA/H	0.703	3	0.134	0.929	45.762	0.597			1		
CoMSIA/SE	0.788	5	0.111	0.951	68.498	0.601	0.520	0.480			
CoMSIA/SHA	0.702	4	0.106	0.956	75.466	0.566	0.245		0.471		0.284
CoMSIA/HDA	0.698	4	0.140	0.922	26.32	0.693			0.664	0	0.336
CoMSIA/SEDA	0.781	5	0.100	0.960	84.796	0.602	0.430	0.388		0	0.182
CoMSIA/SHDA	0.702	4	0.107	0.955	73.679	0.670	0.342		0.408	0.001	0.249
**CoMSIA/SEHDA**	**0.814**	**6**	**0.091**	**0.967**	**102.992**	**0.722**	**0.183**	**0.343**	**0.337**	**0.001**	**0.136**

R² represents the square of the non-cross-validated coefficient.

Q² is the square of the leave-one-out (LOO) cross-validation coefficient.

R²pred denotes the square of the prediction coefficient.

N refers to the optimal number of components.

SEE stands for the standard error of estimation in non-cross-validated analysis.

F is the value obtained from the F-test.

S, E, H, D, and A correspond to the steric, electrostatic, hydrophobic, hydrogen-bond donor, and hydrogen-bond acceptor contributions, respectively.

**Table 4:** Predicted pIC_50_ values and corresponding CoMSIA descriptors for the compounds in this study (*: test set).

Compounds	pIC_50_	pIC_50_ pred	S	E	H	D	A
5a	6.745	6.696	7.92	1.20	4.45	1.42	2.34
5b	6.796	6.901	7.92	1.19	4.50	1.42	2.34
5d	6.620	6.633	7.48	1.08	5.21	1.42	2.34
5e	7.187	7.184	7.50	1.06	6.11	1.42	2.96
5g	6.854	6.998	7.83	1.10	5.48	1.42	2.34
5h	7.268	7.272	8.51	1.11	6.11	1.42	2.33
5i	7.013	6.931	8.58	1.09	6.12	1.43	2.33
5j	7.658	7.569	8.84	1.10	6.44	1.42	2.34
5k	6.745	6.816	8.92	1.10	6.42	1.42	2.34
5l	6.237	6.262	8.99	1.24	6.55	1.42	2.34
5m	7.699	7.704	9.13	1.11	6.72	1.42	2.34
5n	8.222	8.067	9.42	1.10	7.02	1.42	2.34
6a	6.721	6.720	7.60	1.45	5.91	1.42	2.78
6c	6.469	6.474	8.44	1.61	4.94	1.42	2.8
6d	6.523	6.558	8.43	1.60	4.97	1.42	2.79
6e	6.509	6.417	8.03	1.55	5.64	1.42	2.77
6f	6.658	6.651	8.03	1.55	5.63	1.42	2.77
6g	6.602	6.475	8.34	1.53	5.93	1.42	2.77
6h	6.699	6.681	8.35	1.53	5.89	1.42	2.77
6i	6.699	6.668	8.36	1.53	6.95	1.42	2.74
6j	6.721	6.792	9.00	1.54	6.47	1.43	2.74
6k	6.509	6.493	9.06	1.53	6.46	1.42	2.76
6m	7.119	7.163	9.59	1.53	7.07	1.42	2.78
6n	7.602	7.668	9.88	1.53	7.36	1.42	2.77
6o	7.000	7.158	9.23	1.44	7.54	1.42	2.78
6q	6.824	6.697	9.26	1.70	8.42	1.42	2.77
6r	7.000	7.010	9.55	1.71	8.65	1.42	2.77
6s	7.167	7.208	9.83	1.71	8.88	1.42	2.76
5c*	7.367	7.029	7.50	1.08	5.32	1.42	2.34
5f*	7.208	7.049	7.83	1.08	6.65	1.43	2.33
6b*	6.114	6.677	8.02	1.54	5.58	1.42	2.77
6l*	7.886	7.181	9.30	1.53	6.95	1.42	2.78
6p*	6.699	7.085	9.54	1.44	7.95	1.42	2.78

The results in **Table 5** show that the CoMSIA/SEHDA model has met all evaluation criteria and aligns with the standards set by Golbraikh, and Tropsha [30–32]. This model offers a deeper understanding of activity compared to the CoMFA model, with enhanced predictive capabilities for new compounds and adherence to all required validation protocols. Consequently, we used the CoMSIA/SEHDA contour maps to elucidate the structural elements that enhance activity and to aid in the discovery of new active compounds.

**Table 5:** Statistical parameters for validating the CoMSIA/SEHDA model.

Statistical parameter	Score	Threshold	Comment
R^2^_pred_	0.722	More than 0.600	Passed
R_0_^2^: Determination coefficient for the plot of predicted against observed at zero intercept	0.882	More than 0.600	Passed
R_0_^’2^: Determination coefficient of the plot of observed versus predicted at zero intercept.	0.978	More than 0.600	Passed
$\left|R_{0}^{2}-{R^{\prime }}_{0}^{2}\right|$	0.096	Less than 0.300	Passed
$\frac{R^{2}-R_{0}^{2}}{R^{2}}$	-0.221	Less than 0.100	Passed
$\frac{R^{2}-R_{0}^{\prime 2}}{R^{2}}$	-0.354	Less than 0.100	Passed
K: Zero intercept slope of predicted against observed activity for the test set	1.008	0.85 ≤K≤1.15	Passed
K’: Zero intercept slope of observed against predicted activity for the test set	0.987	$0.85\;\leq K^{\prime }\leq 1.15$	Passed

### 3.2. Graphical Analysis of the CoMSIA Model

The CoMSIA contour map visualizes data from the selected 3D-QSAR model, with compound 5n as the reference. **Fig 2** presents the SEHDA model’s contour map for steric, electrostatic, hydrophobic, hydrogen bond donor, and hydrogen bond acceptor fields. In the steric contour maps depicted in **Fig 2A**, green contours (80% contribution) indicate areas where bulkier substitutions enhance biological activity, whereas yellow contours (20% contribution) identify areas where such substitutions diminish activity. The electrostatic maps in **Fig 2B** show blue contours (80% contribution) highlighting regions where positive electrostatic groups are beneficial, and red contours (20% contribution) where negatively charged groups are favored. In the hydrophobic maps, illustrated in **Fig 2C**, yellow contours (80% contribution) point out favorable hydrophobic regions, while white contours (20% contribution) indicate advantageous hydrophilic areas. The hydrogen bond donor maps in **Fig 2D** feature cyan contours (80% contribution), suggesting that hydrogen bond donor groups boost activity, and purple contours (20% contribution) indicate less favorable regions. Lastly, in the hydrogen bond acceptor maps shown in **Fig 2E**, magenta contours (80% contribution) mark areas where hydrogen bond acceptor substitutions enhance activity, and red contours (20% contribution) show where they hinder activity.

### 3.3. Design of new drug candidates

**Fig 3:**
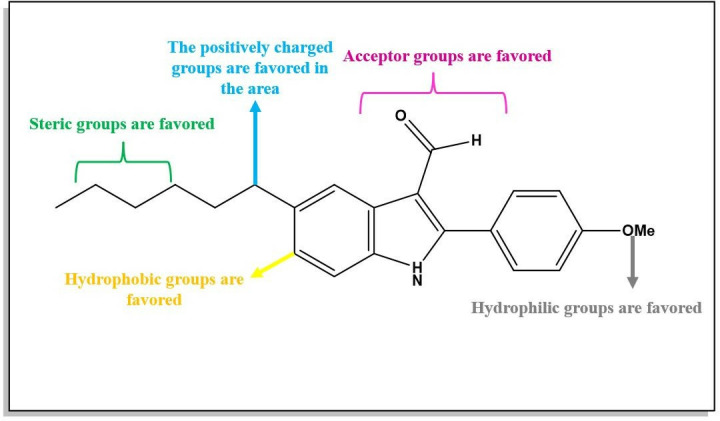
Structure-activity relationship derived from CoMSIA- SEHDA.

**Fig 4:**
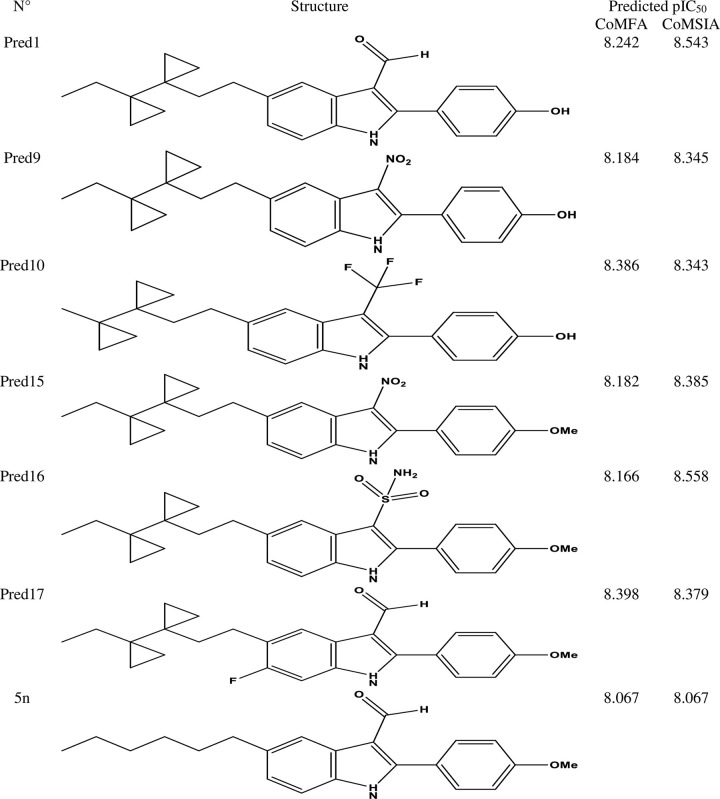
Structures and Predicted pIC_50_ Activities (Pred) According to the 3D-QSAR Model for Predicted Compounds.

The contour map analysis of the CoMSIA models provided a foundation for designing novel inhibitors. Using this analysis, we identified key structural features essential for activity, which guided the optimization process. A summary of the design process is illustrated in **Fig 3**, with compound 5n, the most active compound in the dataset, selected as the template for further modifications (**Fig 3**). The CoMSIA/SEHDA model, which demonstrated superior external validation, was then employed to predict the pIC_50_ values of the newly designed compounds. This approach successfully identified six candidate compounds with predicted activity values exceeding the reference molecule, compound 5n (pIC_50_ = 8.222). The structures and predicted activity values of these compounds are detailed in **Fig 4**, underscoring their potential as promising inhibitors.

### 3.5. ADME and toxicity profiling

The failure of many drug candidates during clinical development is often attributed to poor blood-brain barrier permeability, toxicity, or insufficient efficacy [33]. Therefore, predicting and optimizing the ADMET (Absorption, Distribution, Metabolism, Excretion, and Toxicity) properties of new chemical compounds is critical to avoid complications in the later stages of drug development [34]. This study evaluated the ADMET pharmacokinetic parameters of fifty-nine newly designed compounds using the pkCSM platform (**Table 6**). The results revealed high intestinal absorption rates for the newly designed compounds, ranging from 88.42% to 95.48%, with **Pred17** exhibiting the highest absorption rate suggesting excellent bioavailability.

**Table 6:** ADMET properties of selected molecules including the most active compound.

Ligands	Properties																	
	Absorption	Distribution	Metabolism														Excretion	Toxicity
	Human intestinal absorption	CNS	Cytochrome P450 (CYP450)														Total Clearance	AMES toxicity
					Substrate				Inhibitor									
			2D6		3A4		1A2		2C19		2C9		2D6		3A4			
	(%Absorbed)	log PS		Categorical (Yes/No)													log ml/min/k	Categorical (Yes/No)
**Pred1**	93.16	−1.725		Yes		Yes		No		Yes		Yes		No		No	0.275	Yes
**Pred9**	90.917	−1.787		No		Yes		No		Yes		No		No		No	0.198	No
**Pred10**	88.42	−0.882		Yes		Yes		Yes		Yes		Yes		Yes		No	−0.105	No
**Pred15**	92.796	−1.787		No		Yes		No		No		No		No		No	0.299	No
**Pred16**	94.066	−2.179		No		Yes		Yes		Yes		Yes		No		Yes	0.347	No
**Pred17**	95.486	−0.810		No		Yes		No		Yes		Yes		No		Yes	0.268	No
**5n**	93.287	−0.748		Yes		Yes		Yes		Yes		Yes		No		Yes	0.55	No

Building on these absorption findings, the distribution properties of the compounds were analyzed, particularly their ability to penetrate the central nervous system (CNS) [35]. CNS penetration was evaluated using log PS values, where compounds with a LogPS value greater than −2 are considered effective in crossing the blood-brain barrier [36], while those with a LogPS less than −3 face significant challenges [37]. Among the tested compounds, **Pred16** (−2.179) showed minimal CNS penetration, indicating it is less likely to affect the CNS. In contrast, **5n** (−0.748) exhibited relatively higher permeability, suggesting better potential for CNS penetration.

In addition to distribution, the metabolic profiles of the compounds were assessed, focusing on their interactions with cytochrome P450 (CYP) enzymes. Among the 17 CYP families, 57 CYP genes have been identified in humans, with CYP enzymes playing a crucial role in the biotransformation of approximately 70–80% of clinically used drugs [38]. For this study, the analysis focused on CYP1A2, 2C19, 2D6, 3A4, and 2C9 enzymes. All proposed ligands were identified as substrates for CYP3A4 (**Table 6**), indicating their potential for metabolic processing by this key enzyme.

Following metabolism, the excretion properties of the compounds were evaluated through total clearance values (log ml/min/kg), which ranged from −0.105 (**Pred10**) to 0.55 (**5n**). Ligand **5n**, with the highest clearance value, may have a shorter half-life and could require more frequent dosing. Conversely, **Pred10**, with slower clearance, may provide prolonged therapeutic effects due to its extended presence in the system.

The toxicity profiles of the compounds were assessed to ensure their safety and efficacy. Each compound was evaluated using the AMES test, a widely recognized method for genotoxicity due to its simplicity, cost-effectiveness, and rapid results [39]. As shown in **Table 6**, most of the designed ligands were non-toxic. However, Pred1 exhibited some toxicity, raising concerns for its further development. These findings suggest that while most compounds are promising, certain modifications are needed to improve their non-toxicity, making them strong candidates for clinical evaluation.

### 3.6. Molecular docking study

Molecular docking is central to the design and screening of new bioactive molecules [40–42]. In this study, molecular docking of the three protein targets (CDK2, EGFR, and Tubulin) with all newly designed molecules was performed to identify optimal binding modes that facilitate the inhibition of these targets. Virtual screening was employed to select molecules with the highest binding affinity scores. The docking procedures were validated as outlined in the methodology section, with Root Mean Square Deviation (RMSD) scores ranging from 1.08 to 1.83 Å. An RMSD value below 2.0 Å indicates a reliable predictive protocol for assessing protein-ligand interactions, confirming the docking protocols’ appropriateness [43]. Additionally, parameters such as binding affinity, specific amino acid residues, and grid box dimensions were analyzed during validation. Lower binding affinity values signify stronger interactions between the ligand and the target [54]. **Table 7** presents the binding affinity values for the most favorable interaction poses of all designed molecules with CDK2, EGFR, and Tubulin, using **molecule 5n** and FDA-approved drugs as controls for comparison. All the designed molecules demonstrate superior binding affinity compared to the reference drug and molecule 5n across all selected targets, with Pred9 and Pred10 exhibiting the better binding affinity among the six designed compounds. This suggests that the newly designed compounds could offer enhanced efficacy and stronger target interactions, highlighting their promising therapeutic potential.

**Table 7:** Docking score of the identified compounds against Cyclin-Dependent Kinase 2 (PDB ID: 2A4L), Tubulin (PDB ID: 1AS0), and Epidermal Growth Factor Receptor tyrosine kinase (PDB ID: 1M17) for anti-cancer activity.

Compounds	Binding affinity (kcal/mol)		
CDK2 (2A4L)	Tubulin (1AS0)	EGFR (1M17)
Pred1	−8.7	−8.3	−8.3
Pred9	−9.8	−8.9	−8.2
Pred10	−9.6	−8.5	−8.6
Pred15	−9	−8.2	−8.2
Pred16	−9.2	−8.5	−7.6
Pred17	−9	−8.1	−8.5
5n	−8.5	−7.3	−8.1
Roscovitine	−8.2		
Colchicine		−7.2	
Erlotinib			−7.5

Furthermore, the two-dimensional binding interaction of the compounds (Pred9, Pred10, Pred15, Pred16 and and Pred17), most active molecule 5n, and the reference drug for each protein target showed a similar interaction in the binding pocket of all the targets. This is due to several amino acids participating in the same interactions compared to the FDA drugs and molecule 5n.

**Fig 5:**
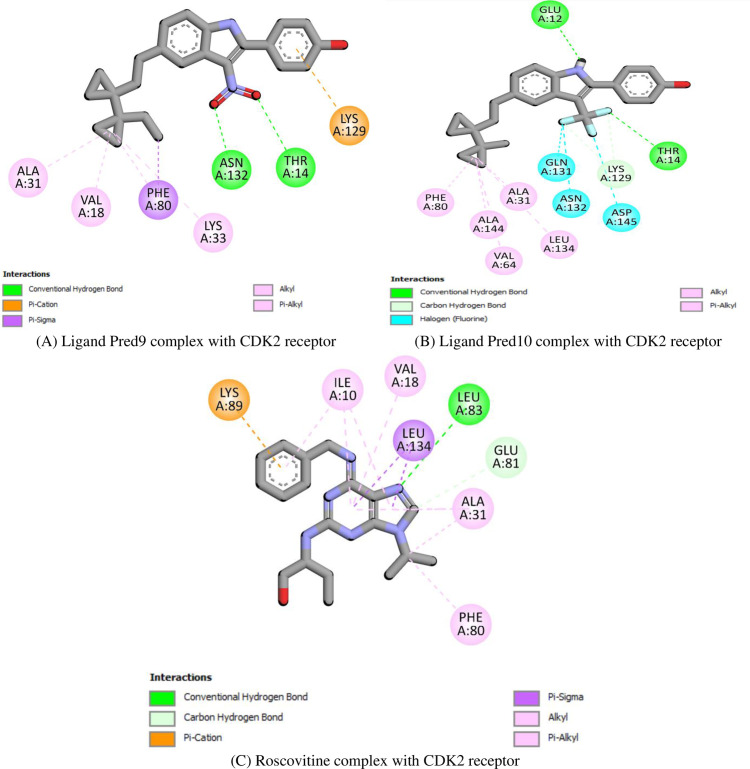
Docking simulation of the interaction between (A) Ligand Pred9, (B) Ligand Pred10 and **(C)** Roscovitine (reference drug) with CDK2 protein.

In the context of CDK2, **Pred9** demonstrates strong binding stability through hydrogen bonds with Asn132 and Thr14, a pi-cation interaction with Lys129, and hydrophobic interactions with Ala31, Val18, and Lys33 (**Fig 5A**). Similarly, **Pred10** forms hydrogen bonds with Glu12 and Thr14, halogen interactions with Gln131, Asp145, and Asn132, and hydrophobic contacts with Ala31, Val64, and Phe80 (**Fig 5B**). For comparison, **Roscovitine**, a known CDK2 inhibitor, exhibits stabilizing interactions such as a pi-cation bond with Lys89, hydrogen bonds with Leu83, and hydrophobic contacts with Ile10 and Val18 (**Fig 5C**)

**Fig 6:**
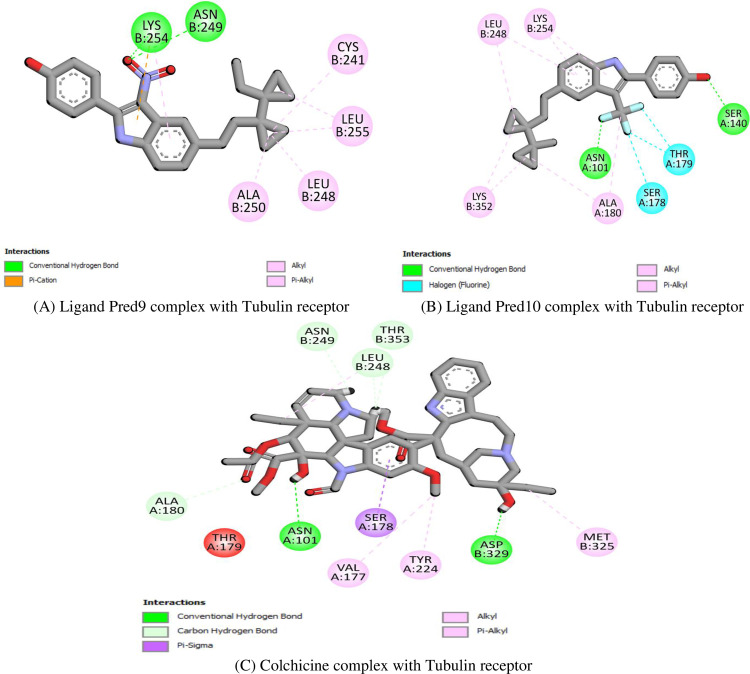
Docking simulation of the interaction between (A) Ligand Pred9, (B) Ligand Pred10 and **(C)** Colchicine (reference drug) with Tubulin protein.

In the case of the Tubulin receptor, **Pred9** forms hydrogen bonds with Lys254 and Asn249, a pi-cation interaction with Lys254, and hydrophobic interactions with Leu248 and Cys241 (**Fig 6A**). Similarly, **Pred10** demonstrates significant binding through hydrogen bonds with Ser140 and Asn101, halogen interactions with Ser178 and Thr179, and hydrophobic contacts with Leu248 and Lys352 (**Fig 6B**). For comparison, **Colchicine**, a well-known Tubulin inhibitor, stabilizes through hydrogen bonds with Asn249 and Thr353, a pi-sigma interaction with Ser178, and hydrophobic contacts with Leu248 and Ala180 (**Fig 6C**)

**Fig 7:**
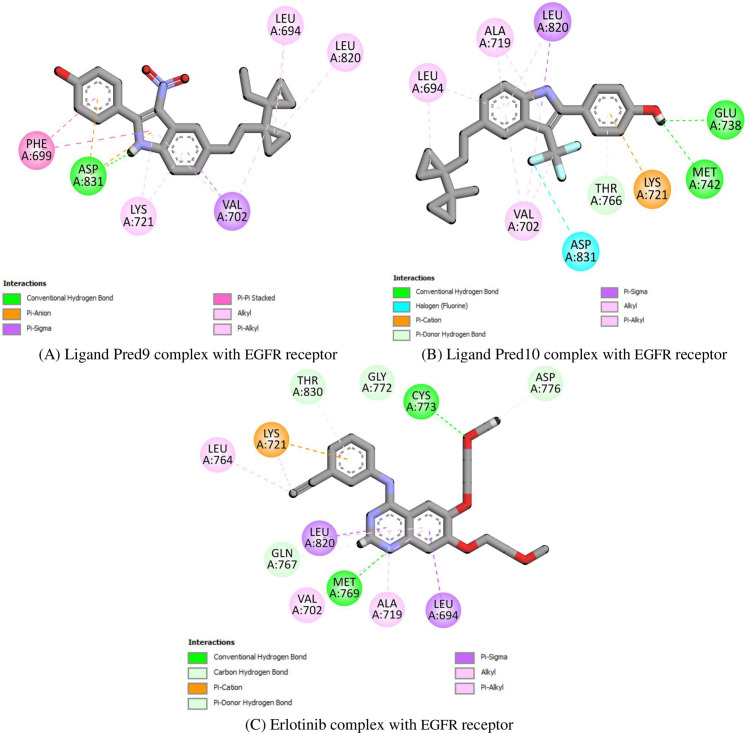
Docking simulation of the interaction between (A) Ligand Pred9, (B) Ligand Pred10 and **(C)** Erlotinib (reference drug) with EGFR protein.

For the EGFR receptor, **Pred9** exhibits stabilizing interactions, including a hydrogen bond and pi-anion interaction with Asp831, pi-sigma interactions with Val702, and hydrophobic contacts with Leu694 and Lys721 (**Fig 7A**). **Pred10** also strongly binds through a halogen bond with Asp831, hydrogen bonds with Glu738 and Met742, and hydrophobic interactions with Val702 and Leu820 (**Fig 7B**). For comparison, **Erlotinib**, an FDA-approved EGFR inhibitor, stabilizes through hydrogen bonds with Met769 and Cys773, a pi-cation interaction with Lys721, and hydrophobic contacts with Val702 and Leu764 (**Fig 7C**). These results emphasize the strong potential of the proposed compounds as multi-target drugs. Research indicates that multi-target compounds are particularly effective in treating complex diseases.

### 3.7. Molecular dynamics (MD)

Molecular dynamics (MD) simulations were conducted to evaluate the stability and dynamic interactions of protein-ligand complexes over time. These simulations modeled interatomic forces and generated trajectories to capture molecular fluctuations, providing detailed insights into interaction dynamics [44]. Specifically, MD simulations were performed for 100 ns on CDK2, EGFR, and Tubulin proteins complexed with Pred9, Pred10, and the most active compound. The resulting trajectories were analyzed to assess system stability and structural properties, including root mean square deviation (RMSD), root mean square fluctuation (RMSF), radius of gyration (Rg), and hydrogen bonding [23].

#### RMSD analysis.

RMSD measures the displacement of a protein’s backbone atoms from their initial positions, providing insights into conformational stability. Lower RMSD values indicate greater stability, reflecting smaller deviations from the starting structure [45].

**Fig 8:**
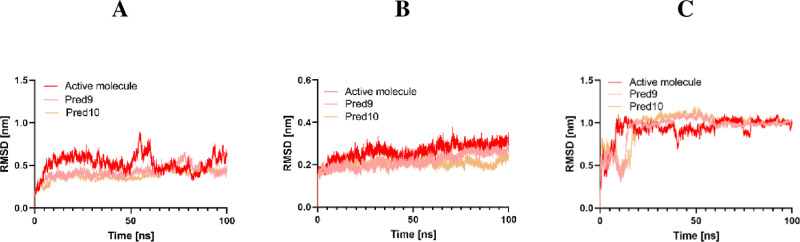
RMSD Analysis of Active Molecules Pred9 and Pred10 in Complex with A: Tubulin, B: CDK2, and C: EGFR.

For the CDK2 complexes, the CDK2/Pred9 and CDK2/Pred10 complexes-maintained equilibrium with RMSD values ranging from 0.12 to 0.25 nm over the 100 ns MD simulation period (**Fig 8**). In contrast, the CDK2/Active molecule complex showed higher fluctuations. Similarly, the EGFR/Active molecule, EGFR/Pred9, and EGFR/Pred10 complexes exhibited comparable trajectories up to 80 ns. Beyond this point, the RMSD values for Pred9 and Pred10 stabilized and converged, demonstrating greater structural stability than the EGFR/Active molecule complex. For Tubulin, the Tubulin/Pred9 and Tubulin/Pred10 complexes achieved stability after 80 ns, while the Tubulin/Active molecule complex remained unstable throughout the simulation.

These results indicate that Pred9 and Pred10 exhibit the least RMSD fluctuations compared to the active compounds, suggesting that these designed compounds enhance target stability by minimizing protein backbone movement. This observation aligns with docking results, which showed better binding affinities for Pred9 and Pred10. However, RMSD alone cannot fully assess system stability, as it does not capture localized fluctuations. To address this limitation, RMSF analysis was performed to evaluate residue-specific variations throughout the trajectory.

#### RMSF analysis.

**Fig 9:**
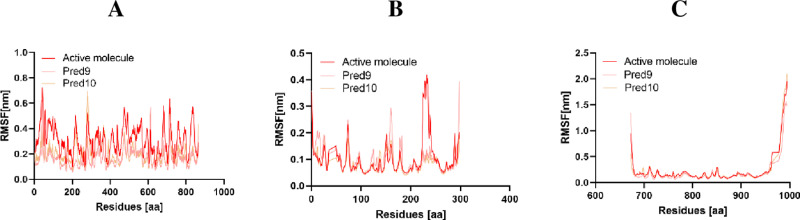
RMSF Analysis of Active Molecule, Pred9, and Pred10 in Complex with A: Tubulin, B: CDK2, and C: EGFR.

RMSF is a crucial parameter in MD simulations used to assess protein flexibility and identify regions with significant structural variations [46]. By calculating RMSF values for each complex, it is possible to determine which residues experience the most pronounced fluctuations during the 100 ns MD simulation (**Fig 9**). Higher RMSF values indicate greater mobility of the protein’s alpha carbon atoms, whereas lower values suggest more stable regions within the protein structure [47].

The designed compounds, Pred9 and Pred10, exhibited lower RMSF values compared to the active compounds when bound to EGFR, CDK2, and Tubulin. This reduced RMSF indicates that individual residues in these proteins experienced fewer atomic variations when complexed with Pred9 and Pred10, resulting in enhanced structural stability. The improved stability suggests that Pred9 and Pred10 form stronger and more consistent interactions with the protein targets, which may translate to more effective inhibition and therapeutic outcomes. These findings align with the RMSD results, where Pred9 and Pred10 also demonstrated lower deviations, indicating minimal backbone movement. The small fluctuations observed for Pred9 and Pred10 further highlight their ability to stabilize residual flexibility, resulting in fewer structural variations compared to the control compounds.

#### Radius of gyration.

**Fig 10:**
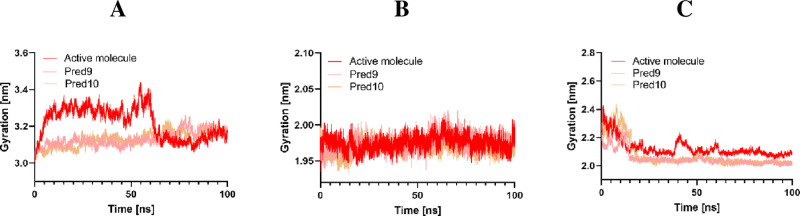
Radius of gyration Analysis of Active Molecule, Pred9, and Pred10 in Complex with A: Tubulin, B: CDK2, and C: EGFR.

The radius of gyration (Rg) measures the compactness of a molecular structure by quantifying the average distance of its atoms from its center of mass [48]. **Fig 10** presents the radius of gyration for all the selected compounds. Compounds 9 and 10 exhibit a lower radius of gyration compared to the most active compounds when interacting with EGFR, CDK2, and Tubulin. This lower radius of gyration indicates a more compact and stable conformation of the protein-ligand complexes, suggesting that compounds 9 and 10 promote tighter binding and a more ordered arrangement within the binding sites. This enhanced compactness is consistent across all three protein targets, reflecting improved stabilization and potentially more effective inhibition

#### Hbond analysis.

**Fig 11:**
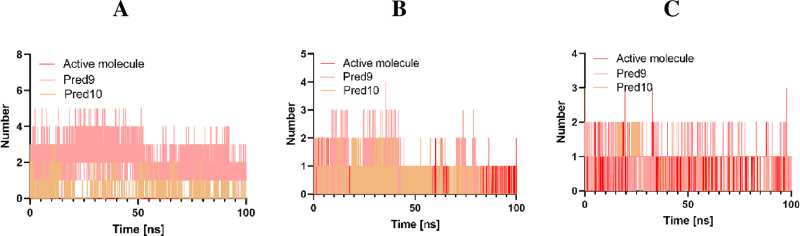
H-bond Analysis of Active Molecule, Pred9, and Pred10 in Complex with A: Tubulin, B: CDK2, and C: EGFR.

Hydrogen bonds play a critical role in molecular recognition by determining the directionality and specificity of interactions between proteins and chemicals [23]. To evaluate the stability of the docked complexes, we analyzed the hydrogen bonds formed by the designed compounds 9 and 10 (**Fig 11**), the most active compound and their target proteins (EGFR, CDK2, and Tubulin) during molecular dynamics (MD) simulations in a solvent environment.

Our calculations reveal that compounds 9 and 10 form a greater number of hydrogen bonds with these proteins compared to the most active compound, indicating more stable interactions. This observation is supported by the RMSD and RMSF plots shown in **Figs 8 and 9**, demonstrating that the designed compounds exhibit tighter binding than the most active compound. These results highlight the potential of compounds 9 and 10 as highly effective inhibitors of EGFR, CDK2, and Tubulin, crucial targets in cancer therapy. By enhancing the binding stability through increased hydrogen bond formation, these compounds could offer a more potent to disrupt tumor cell proliferation.

## 4. Conclusion

In this study, a 3D-QSAR analysis was performed using CoMFA and CoMSIA methods to develop a QSAR model correlating the biological activity of 2-Phenylindole derivatives against the MCF7 breast cancer cell line. The optimized CoMSIA/ SEHDA model demonstrated strong reliability and predictive accuracy, as evidenced by validation metrics (Q² = 0.814, R² = 0.967, R²_pred_ = 0.722). This model’s contour maps designed six novel and enhanced anticancer inhibitors, with ADMET screening confirming their favorable pharmacokinetic profiles. Molecular docking studies revealed that the newly designed compounds exhibited better binding affinities, ranging from −7.2 to −9.8 kcal/mol, to key cancer-related targets (CDK2, EGFR, and Tubulin). Additionally, 100 ns molecular dynamics simulations confirmed the stability of the best-docked complexes within the binding pockets of these targets, highlighting their potential as promising candidates for anticancer drug development. However, it is important to emphasize that the clinical viability and safety of these compounds require further validation through in vitro and in vivo studies.

## Supporting information

S1 Table The selected targets and the coordinates of the grid box. (DOCX)

## References

[1] Nagai H, Kim YH. Cancer prevention from the perspective of global cancer burden patterns. J Thorac Dis. 2017;9(3):448–51. doi: 10.21037/jtd.2017.02.75 28449441 PMC5394024

[2] Ryszkiewicz P, Malinowska B, Schlicker E. Polypharmacology: promises and new drugs in 2022. Pharmacol Rep. 2023;75(4):755–70. doi: 10.1007/s43440-023-00501-4 37278927 PMC10243259

[3] Talevi A. Multi-target pharmacology: possibilities and limitations of the “skeleton key approach” from a medicinal chemist perspective. Front Pharmacol. 2015;6:205. doi: 10.3389/fphar.2015.00205 26441661 PMC4585027

[4] Hawash M. Highlights on specific biological targets; cyclin-dependent kinases, epidermal growth factor receptors, Ras protein, and cancer stem cells in anticancer drug development. Anticancer Drug Development. 2019. p. 471–8. 10.1055/a-0898-7347 31083741

[5] Pellarin I, Dall’Acqua A, Favero A, Segatto I, Rossi V, Crestan N, et al. Cyclin-dependent protein kinases and cell cycle regulation in biology and disease. Signal Transduct Target Ther. 2025;10(1):11. doi: 10.1038/s41392-024-02080-z 39800748 PMC11734941

[6] Tomuleasa C, Tigu A-B, Munteanu R, Moldovan C-S, Kegyes D, Onaciu A, et al. Therapeutic advances of targeting receptor tyrosine kinases in cancer. Signal Transduct Target Ther. 2024;9(1):201. doi: 10.1038/s41392-024-01899-w 39138146 PMC11323831

[7] Lopes D, Maiato H. The Tubulin Code in Mitosis and Cancer. Cells. 2020;9(11):2356. doi: 10.3390/cells9112356 33114575 PMC7692294

[8] El-Sawy ER, Bassyouni FA, Abu-Bakr SH, Rady HM, Abdlla MM. Synthesis and biological activity of some new 1-benzyl and 1-benzoyl-3-heterocyclic indole derivatives. Acta Pharm. 2010;60(1):55–71. doi: 10.2478/v10007-010-0004-0 20228041

[9] Golob T, Liebl R, von Angerer E. Sulfamoyloxy-substituted 2-phenylindoles: antiestrogen-based inhibitors of the steroid sulfatase in human breast cancer cells. Bioorg Med Chem. 2002;10(12):3941–53. doi: 10.1016/s0968-0896(02)00306-1 12413846

[10] Trogden BG, Kim SH, Lee S, Katzenellenbogen JA. Tethered indoles as functionalizable ligands for the estrogen receptor. Bioorg Med Chem Lett. 2009;19(2):485–8. doi: 10.1016/j.bmcl.2008.11.043 19059778 PMC2680426

[11] Gastpar R, Goldbrunner M, Marko D, von Angerer E. Methoxy-substituted 3-formyl-2-phenylindoles inhibit tubulin polymerization. J Med Chem. 1998;41(25):4965–72. doi: 10.1021/jm980228l 9836614

[12] Pojarová M, Kaufmann D, Gastpar R, Nishino T, Reszka P, Bednarski PJ, et al. [(2-Phenylindol-3-yl)methylene]propanedinitriles inhibit the growth of breast cancer cells by cell cycle arrest in G(2)/M phase and apoptosis. Bioorg Med Chem. 2007;15(23):7368–79. doi: 10.1016/j.bmc.2007.07.046 17889547

[13] Ezelarab HAA, Ali TFS, Abbas SH, Sayed AM, Beshr EAM, Hassan HA. New antiproliferative 3-substituted oxindoles inhibiting EGFR/VEGFR-2 and tubulin polymerization. Mol Divers. 2023;28(2):563–80. doi: 10.1007/s11030-023-10603-z 36790582 PMC11070402

[14] Devi N, Kaur K, Biharee A, Jaitak V. Recent Development in Indole Derivatives as Anticancer Agent: A Mechanistic Approach. Anticancer Agents Med Chem. 2021;21(14):1802–24. doi: 10.2174/1871520621999210104192644 33397272

[15] Kaufmann D, Pojarová M, Vogel S, Liebl R, Gastpar R, Gross D, et al. Antimitotic activities of 2-phenylindole-3-carbaldehydes in human breast cancer cells. Bioorg Med Chem. 2007;15(15):5122–36. doi: 10.1016/j.bmc.2007.05.030 17533132

[16] Clark M, Cramer RD III, Van Opdenbosch N. Validation of the general purpose tripos 5.2 force field. J Comput Chem. 1989;10(8):982–1012. doi: 10.1002/jcc.540100804

[17] Purcell WP, Singer JA. A brief review and table of semiempirical parameters used in the Hueckel molecular orbital method. J Chem Eng Data. 1967;12(2):235–46. doi: 10.1021/je60033a020

[18] Khatabi KEL, Aanouz I, Khaldan A, Ajana MA, Lakhlifi T. 3D-Qsar and Molecular Docking Analysis of the in Vitro β-Glucuronidase Inhibitory Activity of N-Benzyl Deoxynojirimycin Derivatives. 2020;3:89–98.

[19] Hennebed PA. E. T. Validation of a. 1996;193:134–42.

[20] Khatabi KE, El-mernissi R, Moukhliss Y, Hajji H, Rehman HM, Yadav R, et al. Rational design of novel potential EGFR inhibitors by 3D-QSAR, molecular docking, molecular dynamics simulation, and pharmacokinetics studies. Chemical Data Collections. 2022;39:100851. doi: 10.1016/j.cdc.2022.100851

[21] Sherin DR, Geethu CK, Prabhakaran J, Mann JJ, Dileep Kumar JS, Manojkumar TK. Molecular docking, dynamics simulations and 3D-QSAR modeling of arylpiperazine derivatives of 3,5-dioxo-(2H,4H)-1,2,4-triazine as 5-HT1AR agonists. Comput Biol Chem. 2019;78:108–15. doi: 10.1016/j.compbiolchem.2018.11.015 30502727

[22] Pettersen EF, Goddard TD, Huang CC, Couch GS, Greenblatt DM, Meng EC, et al. UCSF Chimera--a visualization system for exploratory research and analysis. J Comput Chem. 2004;25(13):1605–12. doi: 10.1002/jcc.20084 15264254

[23] Baammi S, El Allali A, Daoud R. Unleashing Nature’s potential: a computational approach to discovering novel VEGFR-2 inhibitors from African natural compound using virtual screening, ADMET analysis, molecular dynamics, and MMPBSA calculations. Front Mol Biosci. 2023;10:1227643. doi: 10.3389/fmolb.2023.1227643 37800126 PMC10548200

[24] Prasada Rao CM, Silakabattini K, Narapusetty N, Marabathuni VJP, Thejomoorthy K, Rajeswari T, et al. Insights from the molecular docking and simulation analysis of P38 MAPK phytochemical inhibitor complexes. Bioinformation. 2023;19(3):323–30. doi: 10.6026/97320630019323 37808383 PMC10557437

[25] Al-Khafaji K, Taskin Tok T. Molecular dynamics simulation, free energy landscape and binding free energy computations in exploration the anti-invasive activity of amygdalin against metastasis. Comput Methods Programs Biomed. 2020;195:105660. doi: 10.1016/j.cmpb.2020.105660 32726718

[26] Baammi S, El Allali A, Daoud R. Potent VEGFR-2 inhibitors for resistant breast cancer: a comprehensive 3D-QSAR, ADMET, molecular docking and MMPBSA calculation on triazolopyrazine derivatives. Front Mol Biosci. 2023;10:1288652. doi: 10.3389/fmolb.2023.1288652 38074087 PMC10702966

[27] Mark P, Nilsson L. Structure and Dynamics of the TIP3P, SPC, and SPC/E Water Models at 298 K. J Phys Chem A. 2001;105(43):9954–60. doi: 10.1021/jp003020w

[28] Baammi S, Daoud R, El Allali A. In silico protein engineering shows that novel mutations affecting NAD+ binding sites may improve phosphite dehydrogenase stability and activity. Sci Rep. 2023;13(1). doi: 10.1038/s41598-023-28246-3 PMC9892502 36725973

[29] En-Nahli F, Baammi S, Hajji H, Alaqarbeh M, Lakhlifi T, Bouachrine M. High-throughput virtual screening approach of natural compounds as target inhibitors of plasmepsin-II. J Biomol Struct Dyn. 2023;41(19):10070–80. doi: 10.1080/07391102.2022.2152871 36469727

[30] Moussaoui M, Baassi M, Baammi S, Soufi H, Salah M, Daoud R, et al. In silico design of novel CDK2 inhibitors through QSAR, ADMET, molecular docking and molecular dynamics simulation studies. J Biomol Struct Dyn. 2023;41(23):13646–62. doi: 10.1080/07391102.2023.2212304 37203327

[31] Baassi M, Moussaoui M, Soufi H, Rajkhowa S, Sharma A, Sinha S, et al. Towards designing of a potential new HIV-1 protease inhibitor using QSAR study in combination with Molecular docking and Molecular dynamics simulations. PLoS One. 2023;18(4):e0284539. doi: 10.1371/journal.pone.0284539 37079533 PMC10118106

[32] Soufi H, Moussaoui M, Baammi S, Baassi M, Salah M, Daoud R, et al. Multi-combined QSAR, molecular docking, molecular dynamics simulation, and ADMET of Flavonoid derivatives as potent cholinesterase inhibitors. Journal of Biomolecular Structure and Dynamics. 2023;42(12):6027–41. doi: 10.1080/07391102.2023.2238314 37485860

[33] Moussaoui M, Baammi S, Soufi H, Baassi M, Salah M, Allali AE, et al. Design and Optimization of Quinazoline Derivatives as Potent EGFR Inhibitors for Lung Cancer Treatment: A Comprehensive QSAR, ADMET, and Molecular Modeling Investigation. ACS Omega. 2024;9(46):45842–57. doi: 10.1021/acsomega.4c04639 39583714 PMC11579736

[34] Vrbanac J, Slauter R. Overview of ADME Science. A Comprehensive Guide to Toxicology in Nonclinical Drug Development. Elsevier. 2024. p. 49–82. doi: 10.1016/b978-0-323-85704-8.00010-4

[35] Elbouhi M, Ouabane M, Tabti K, Badaoui H, Abdessadak O, El Alaouy MA, et al. Computational evaluation of 1,2,3-triazole-based VEGFR-2 inhibitors: anti-angiogenesis potential and pharmacokinetic assessment. J Biomol Struct Dyn. 2025;43(5):2549–59. doi: 10.1080/07391102.2023.2301686 38193897

[36] Moussaoui M, Baammi S, Soufi H, Baassi M, El Allali A, Belghiti ME, et al. QSAR, ADMET, molecular docking, and dynamics studies of 1,2,4-triazine-3(2H)-one derivatives as tubulin inhibitors for breast cancer therapy. Sci Rep. 2024;14(1):16418. doi: 10.1038/s41598-024-66877-2 39013949 PMC11252338

[37] Koubi Y, Moukhliss Y, Hajji H, Abdessadak O, Alaqarbeh M, Ajana MA, et al. Computational structure – biological activity and retrosynthesis investigations of 1,2,3-triazole-quinoline hybrid molecules as potential respiratory virus inhibitors. Chem Heterocycl Comp. 2024;60(9–10):491–504. doi: 10.1007/s10593-024-03367-3

[38] Zhao M, Ma J, Li M, Zhang Y, Jiang B, Zhao X, et al. Cytochrome P450 Enzymes and Drug Metabolism in Humans. Int J Mol Sci. 2021;22(23):12808. doi: 10.3390/ijms222312808 34884615 PMC8657965

[39] Karoui S, Khaoua O, Benbellat N, Antonczak S, Messaoudi A. Molecular Docking, Molecular Dynamics, pkCSM Drug‐Likeness Profiles, Toxicity, and DFT Study of the Antioxidant and Anticancer Activities of Three Flavonoid Derivatives. ChemistrySelect. 2024;9(40). doi: 10.1002/slct.202401776

[40] Agu PC, Afiukwa CA, Orji OU, Ezeh EM, Ofoke IH, Ogbu CO, et al. Molecular docking as a tool for the discovery of molecular targets of nutraceuticals in diseases management. Sci Rep. 2023;13(1):13398. doi: 10.1038/s41598-023-40160-2 37592012 PMC10435576

[41] Elbouhi M, Tabti K, Ouabane M, Alaqarbeh M, Elkamel K, Lakhlifi T, et al. A computational exploration of the antioxidant potential of conjugated quinazolinone Schiff bases. Chem Heterocycl Comp. 2024;60(11–12):627–38. doi: 10.1007/s10593-025-03386-8

[42] Elbouhi M, Tabti K, Elkamel K, Lakhlifi T, Sbai A, Bouachrine M. Novel chemical compounds based on 1,2,3-triazoles as potential Mycobacterium tuberculosis inhibitors. In-silico study through pharmacophore modeling, molecular docking, molecular dynamics, and ADMET predictions. Phys Chem Res. 2025;13(1):11–29. doi: 10.22036/pcr.2024.449578.2507

[43] Ramírez D, Caballero J. Is It Reliable to Take the Molecular Docking Top Scoring Position as the Best Solution without Considering Available Structural Data?. Molecules. 2018;23(5):1038. doi: 10.3390/molecules23051038 29710787 PMC6102569

[44] Saurabh S, Sivakumar PM, Perumal V, Khosravi A, Sugumaran A, Prabhawathi V. Molecular Dynamics Simulations in Drug Discovery and Drug Delivery. Engineering Materials. Springer International Publishing. 2020. p. 275–301. doi: 10.1007/978-3-030-36260-7_10

[45] Liu K, Watanabe E, Kokubo H. Exploring the stability of ligand binding modes to proteins by molecular dynamics simulations. J Comput Aided Mol Des. 2017;31(2):201–11. doi: 10.1007/s10822-016-0005-2 28074360

[46] Song X, Bao L, Feng C, Huang Q, Zhang F, Gao X, et al. Accurate Prediction of Protein Structural Flexibility by Deep Learning Integrating Intricate Atomic Structures and Cryo-EM Density Information. Nat Commun. 2024;15(1). doi: 10.1038/s41467-024-49858-x PMC11219796 38956032

[47] Luo H, Ma Y, Su Z, Gu Y, Zhang S, Gerstweiler L. Investigating the stability of chimeric murine polyomavirus VP1 Capsomeres via molecular dynamics simulations and experimental analysis. Int J Biol Macromol. 2025;286:138372. doi: 10.1016/j.ijbiomac.2024.138372 39643186

[48] Rampogu S, Lee G, Park JS, Lee KW, Kim MO. Molecular Docking and Molecular Dynamics Simulations Discover Curcumin Analogue as a Plausible Dual Inhibitor for SARS-CoV-2. Int J Mol Sci. 2022;23(3):1771. doi: 10.3390/ijms23031771 35163692 PMC8836015

