# Supplementary material for: Multitarget inhibition of CDK2, EGFR, and tubulin by phenylindole derivatives: Insights from 3D-QSAR, molecular docking, and dynamics for cancer therapy
Source: PLoS One. 2025 Jun 17;20(6):e0326245. doi: 10.1371/journal.pone.0326245 (PMC12233951; doi:10.1371/journal.pone.0326245)
Supplement: S1 Table — (DOCX) [file pone.0326245.s001.docx]

***Table S1.*** *The selected targets and the coordinates of the grid box*

| Protein | PDBID | Grid box center (Å) | Grid box size(Å) |
| --- | --- | --- | --- |
| Tubulin | 1SA0 | center_x = 117.219  center_y = 90.179  center_z = 6.289 | size_x = 20  size_y = 18  size_z = 40 |
| Epidermal growth factor Receptor (EGFR) | 1M17 | center_x = 21.697  center_y = 0.303  center_z = 52.093 | size_x = 42  size_y = 18  size_z = 22 |
| Cyclin-dependent kinase (CDK2) | 2A4L | center_x = 100.865  center_y = 101.746  center_z = 79.893 | size_x = 40  size_y = 40  size_z = 40 |
