# Supplementary material for: Multitarget inhibition of CDK2, EGFR, and tubulin by phenylindole derivatives: Insights from 3D-QSAR, molecular docking, and dynamics for cancer therapy
Source: PLoS One. 2025 Jun 17;20(6):e0326245. doi: 10.1371/journal.pone.0326245 (PMC12233951; doi:10.1371/journal.pone.0326245)
Supplement: Attachment — Submitted filename: Multi_PONE-D-24-50354_reviewer.pdf [file pone.0326245.s002.pdf]

Reviewer Comments

Manuscript title: Multitarget Inhibition of CDK2, EGFR, and Tubulin by [(2-Phenylindol-3-yl)methylene] and 2-Phenylindole-3-Carbaldehyde Derivatives: A 3D-QSAR, Molecular Docking, and Molecular Dynamics Approach for Cancer Therapy
The title is very long:
Suggestion: *Multitarget Inhibition of CDK2, EGFR, and Tubulin by Phenylindole Derivatives: Insights from 3D-QSAR, Molecular Docking, and Dynamics for Cancer Therapy*

Introduction

I believe the section could benefit from a clearer discussion of how this study uniquely contributes to the existing body of knowledge. While polypharmacology is highlighted as a promising therapeutic strategy, there is little detail on the specific challenges or gaps in current approaches that this study aims to address. Providing a concise summary of these challenges would contextualize the importance of the study and justify the focus on [(2-Phenylindol-3-yl)methylene]propanedinitrile and 2-phenylindole-3-carbaldehyde derivatives.

Additionally, the introduction could better connect the rationale for using these derivatives to the specific structural or mechanistic advantages they might offer in targeting CDK2, EGFR, and Tubulin. While the significance of these targets is well-articulated, the rationale for their simultaneous inhibition is not thoroughly discussed. Expanding on the potential synergy or therapeutic benefit of multitarget inhibition, and referencing relevant literature, would strengthen the argument and provide a more robust foundation for the study.

Materials and Method

I noticed several critical areas that require more detail for reproducibility. For instance, the molecular docking and dynamics protocols lack key parameters such as grid dimensions, solvent conditions, and scoring functions used during docking. Similarly, the rationale for selecting specific parameters (e.g., the 1.0 nm box size or 100 ns simulation time) is missing. Including this information would make the study more transparent and allow others to replicate the work.

I also feel that the description of the CoMSIA modeling could be expanded. While the statistical metrics are mentioned, there is no discussion of how they compare to similar studies or why the SEHDA model was ultimately chosen. Moreover, the dataset preparation and validation steps for the QSAR models could be more detailed, particularly the choice of training and test sets and any external validation strategies used.

Results and Discussion

The quality of the figures, particularly Figures 1 through 3, is notably poor, which significantly hinders the clarity and interpretation of the results. I strongly recommend that the authors replace all low-resolution figures with high-resolution, clear images that effectively convey the information. Improving the quality of the figures will not only enhance readability but also ensure that the results are presented in a professional and comprehensible manner, meeting the standards expected for publication.

The presentation of the results could benefit from a more integrated discussion that links findings across the different methodologies. For instance, the CoMSIA results identify key structural features contributing to biological activity, but the narrative does not adequately connect these features to the docking and MD simulation outcomes. Establishing this link would provide a more cohesive understanding of how these compounds achieve their multitarget efficacy and stability.

While the emphasis on T09 and T10 is justified by their superior performance, the discussion does not adequately explore the broader implications of multitarget inhibition. For example, the potential for synergistic effects across

CDK2, EGFR, and Tubulin pathways or challenges such as off-target effects and drug resistance is not thoroughly addressed. Moreover, the results section could highlight how these findings compare to existing inhibitors or similar studies, which would contextualize the significance of the compounds' performance. Including such comparisons and discussing the translational potential of T09 and T10, alongside their possible limitations, would enrich the narrative and provide a more balanced perspective.

Conclusion

I feel that the claims about the compounds' potential for clinical development are slightly overstated, as they are based solely on computational data. Including a statement about the need for experimental validation would provide a more balanced perspective and align the conclusion with the study's scope.

Additionally, while the conclusion emphasizes the promising nature of T09 and T10, it does not clearly articulate what sets this study apart from previous research. Highlighting any unique insights or novel contributions to the field would strengthen the impact of the conclusion and provide a compelling case for publication.

Recommendation

After a thorough evaluation of the manuscript, I recommend major revisions before it can be considered for publication. The study is well-conceived, exploring the potential of [(2-Phenylindol-3-yl)methylene]propanedinitrile and 2-phenylindole-3-carbaldehyde derivatives as multitarget inhibitors for cancer therapy. The integration of 3D-QSAR modeling, molecular docking, and molecular dynamics simulations provides a robust computational framework, and the findings are relevant to the development of multitarget anticancer agents, particularly in targeting CDK2, EGFR, and Tubulin. However, some key issues must be addressed to enhance the quality of the manuscript. These include providing greater detail on methodological parameters for reproducibility, improving the quality and resolution of figures to enhance data interpretation, and ensuring a more cohesive integration of results across methodologies. The discussion should delve deeper into the biological implications of multitarget inhibition and address potential limitations, such as the need for experimental validation. Additionally, overstatements regarding the compounds' therapeutic potential should be tempered, with language reflecting the preliminary nature of computational findings. **If these revisions are thoroughly addressed, this study has the potential to make a valuable contribution to the field of cancer drug discovery.**
