# Supplementary material for: Multitarget inhibition of CDK2, EGFR, and tubulin by phenylindole derivatives: Insights from 3D-QSAR, molecular docking, and dynamics for cancer therapy
Source: PLoS One. 2025 Jun 17;20(6):e0326245. doi: 10.1371/journal.pone.0326245 (PMC12233951; doi:10.1371/journal.pone.0326245)
Supplement: Attachment — Submitted filename: REVIEWER REPONSE PLOS ONE 5.odt [file pone.0326245.s004.odt]

Dear Editor,
We would like to thank you and the reviewers for the time and consideration you have dedicated to our manuscript. Your valuable feedback will be a great resource to further refine our work. We wish to acknowledge the reviewers for their comments.
In this revised version of the manuscript, we addressed all the comments and remarks given to us. All changes made to the manuscript are highlighted in yellow. Also, detailed responses to the reviewer’s comments are given below.
We are at your disposal for any additional information or queries, and we are keen to receive your reply.
On behalf of my colleagues/co-authors,  
Sincerely,

 Mohamed Moussaoui


Comments and list of responses:
Reviewer #1:
The manuscript titled "Multitarget Inhibition of CDK2, EGFR, and Tubulin by [(2-Phenylindol-3-yl)methylene] and 2-Phenylindole-3-Carbaldehyde Derivatives: A 3D-QSAR, Molecular Docking, and Molecular Dynamics Approach for Cancer Therapy" explores the use of in-silico methods such as D3-QSAR modeling, molecular docking and Molecular Dynamics (MD) simulations to identify promising cancer therapuetic targets with activities against CDK2, EGFR and Tubulin.
The authors utilized SEHDA CoMSIA model to investigate different combinations of parameters for the targets and tested these using standardized methods. The statistical methods used were appropriate and rigorous. The results were presented in an intelligible fashion and written in standard English.
Molecular docking analysis and molecular dynamics were conducted using standardized methods.
We sincerely appreciate your positive and thoughtful feedback on our manuscript. Your encouraging words strengthen our confidence in the study and inspire us to continue advancing in this field. Thank you for recognizing our efforts and approach.
Reviewer #2:
Thank you for your valuable feedback on our manuscript. We greatly appreciate the time and effort you have taken to provide us with such a thoughtful and thorough evaluation. Your comments have been invaluable in enhancing the accuracy of our work. We have taken each of your remarks into careful consideration and addressed them in detail. Below, we have provided our responses to each of your queries.
Summary and strength

The manuscript investigates the multitarget inhibitory potential of two classes of chemical compounds—methylene]propanedinitrile and 2-phenylindole-3-carbaldehyde derivatives—against three significant cancer-associated proteins: Cyclin-Dependent Kinase 2 (CDK2), Epidermal Growth Factor Receptor (EGFR), and Tubulin. The study employs various in silico techniques, including 3D-QSAR modeling, molecular docking, and molecular dynamics simulations, to evaluate the inhibitory effects of these compounds.
Findings highlight that compounds T09 and T10 demonstrated superior binding affinities and stability across all targeted proteins when compared to the most active compound in the dataset and standard reference drugs. Moreover, the SEHDA CoMSIA model showed strong predictive performance, indicating that steric, electrostatic, hydrophobic, and hydrogen bonding interactions significantly contributed to the activity of these compounds. The results suggest that [(2-Phenylindol-3-yl)methylene]propanedinitrile and 2-phenylindole-3-carbaldehyde derivatives could be a promising new class of multitarget anticancer agents, offering a potential path for further development in cancer treatment strategies.
Limitations
While the SEHDA CoMSIA model demonstrated strong predictive performance, the accuracy of these models may be influenced by the quality and quantity of input data. A large input dataset is highly recommended.
We acknowledge that the SEHDA CoMSIA model’s predictive power relies heavily on the quality and quantity of input data. While our study utilized a carefully curated dataset of 33 compounds (split into training and test sets), we agree that expanding the dataset could enhance the model's robustness. Future work will focus on incorporating a larger dataset of derivatives to strengthen the predictive accuracy and validate the current findings.
The authors are encouraged to perform, at minimum, an in silico-based ADME and toxicity profile of the lead compounds.
We agree with the reviewer that ADME (Absorption, Distribution, Metabolism, and Excretion) and toxicity predictions are critical for identifying lead compounds. We have now performed in silico ADME and toxicity profiling for Pred9 and Pred10 using the SwissADME and pkcsm platforms. The results, which indicate favorable pharmacokinetic and toxicity profiles for these compounds, have been added to the revised manuscript under a new section titled “ADME and Toxicity Profiling”.
A detailed explanation of the mechanism of action for the three selected targets in cancer progression should be provided.
Thank you for your valuable suggestion. We are pleased to inform you that a detailed explanation of the roles of CDK2, EGFR, and Tubulin in cancer progression has been included in the revised manuscript under the section titled "Mechanism of Action for Target Proteins." This section provides a comprehensive discussion of their involvement in cell cycle regulation, proliferation, and structural integrity, contextualizing their selection as therapeutic targets.
The assumption that interactions between the lead compounds and target proteins indicate inhibition should be clarified. The authors should explain the rationale behind this assumption. Could the binding and interactions potentially lead to activation rather than inhibition of the protein targets? If not, why? Additionally, the authors should discuss the relevance and implications of the amino acids involved in binding at the protein's binding pocket. Are these amino acids part of the catalytic or regulatory site?
Thank you for your insightful comment. In response to your suggestion, we have clarified the rationale behind the assumption that interactions between the lead compounds and target proteins indicate inhibition. Specifically, we targeted the same binding pockets as those occupied by the reference drug, ensuring that our compounds interact with the relevant sites known to mediate the inhibitory effects of these targets. This approach helps us confidently assume that our compounds are likely to inhibit the proteins, as we have verified that they interact with the same binding regions as the reference drug.
Additionally, we compared the interactions of the designed molecules with the reference drug to assess whether they target the same amino acids. This comparison ensures that our compounds bind similarly to the reference drug and thus may exhibit similar inhibitory effects.
The labels for Figures 7, 8, 9, and 10 lack clarity. The authors should indicate which protein target corresponds to each figure.
Thank you for your valuable feedback. In response to your suggestion, we have revised the labels for Figures 7, 8, 9, and 10 to clearly indicate which protein target corresponds to each figure.
The term "Lower binding affinity" has been used to describe strong interactions. The authors should differentiate between low and high binding affinity in relation to kcal/mol and revise the text accordingly.
Thank you for your thoughtful suggestion. The text has been revised accordingly to avoid any confusion and ensure the correct interpretation of the binding affinity data.
In Figure 3, the word "Accepteur" should be corrected to the English term.
Thank you for pointing that out. I'll make sure to correct "Accepteur" to the English term "Acceptor" in Figure 3.
The authors referenced Table XX in their discussion, but no such table is available. This should be addressed.
Thank you for bringing this issue to our attention. We corrected in the revised manuscript. The placeholder reference to "Table XX" has been replaced with the appropriate reference to Table 7, ensuring consistency and clarity throughout the text.
Similarly, references to a Supplementary file should be revised if no such file exists.
Thank you for the feedback. I will review the manuscript and revise any references to the Supplementary file to ensure they align with the available materials.
Experimental validation in biological systems is crucial to confirm the efficacy and safety of the identified compounds. By acknowledging these limitations, the authors should emphasize the need for follow-up studies involving empirical validation and testing in biological systems to further evaluate the potential of the identified compounds as anticancer agents.
Thank you for your helpful suggestion. In response, we have revised the Conclusion section to include a limitation on future directions, outlining potential areas for further research. Additionally, we have thoroughly run a grammatical check on the manuscript to ensure clarity and improve readability.
Reviewer #3:
After a thorough evaluation of the manuscript, I recommend major revisions before it can be considered for publication. The study is well-conceived, exploring the potential of [(2-Phenylindol-3-yl)methylene]propanedinitrile and 2-phenylindole-3-carbaldehyde derivatives as multitarget inhibitors for cancer therapy. The integration of 3D-QSAR modeling, molecular docking, and molecular dynamics simulations provides a robust computational framework, and the findings are relevant to the development of multitarget anticancer agents, particularly in targeting CDK2, EGFR, and Tubulin. However, some key issues must be addressed to enhance the quality of the manuscript. These include providing greater detail on methodological parameters for reproducibility, improving the quality and resolution of figures to enhance data interpretation, and ensuring a more cohesive integration of results across methodologies. The discussion should delve deeper into the biological implications of multitarget inhibition and address potential limitations, such as the need for experimental validation. Additionally, overstatements regarding the compounds’ therapeutic potential should be tempered, with language reflecting the preliminary nature of computational findings. If these revisions are thoroughly addressed, this study has the potential to make a valuable contribution to the field of cancer drug discovery. Detailed comments are attached as PDF
Thank you for your valuable feedback on our manuscript. We greatly appreciate the time and effort you have taken to provide us with such a thoughtful and thorough evaluation. Your comments have been invaluable in enhancing the accuracy of our work. We have taken each of your remarks into careful consideration and addressed them in detail. Below, we have provided our responses to each of your queries.
Title
Comment: The title is very long.
Suggestion: Multitarget Inhibition of CDK2, EGFR, and Tubulin by Phenylindole Derivatives: Insights from 3D-QSAR, Molecular Docking, and Dynamics for Cancer Therapy.
Thank you for your suggestion. The title has been revised to:
"Multitarget Inhibition of CDK2, EGFR, and Tubulin by Phenylindole Derivatives: Insights from 3D-QSAR, Molecular Docking, and Dynamics for Cancer Therapy."
This updated title is more concise and aligns with your recommendation.
Introduction
I believe the section could benefit from a clearer discussion of how this study uniquely contributes to the existing body of knowledge. While polypharmacology is highlighted as a promising therapeutic strategy, there is little detail on the specific challenges or gaps in current approaches that this study aims to address. Providing a concise summary of these challenges would contextualize the importance of the study and justify the focus on [(2-Phenylindol-3- yl)methylene]propanedinitrile and 2-phenylindole-3-carbaldehyde derivatives.
Additionally, the introduction could better connect the rationale for using these derivatives to the specific structural or mechanistic advantages they might offer in targeting CDK2, EGFR, and Tubulin. While the significance of these targets is well-articulated, the rationale for their simultaneous inhibition is not thoroughly discussed. Expanding on the potential synergy or therapeutic benefit of multitarget inhibition, and referencing relevant literature, would strengthen the argument and provide a more robust foundation for the study.
Thank you for your insightful comments. We appreciate your suggestions for clarifying the unique contributions of our study.
In response, we have revised the introduction to more clearly highlight the specific challenges in current polypharmacology approaches, particularly the limited success in rationally designing multitarget inhibitors that can effectively modulate diverse oncogenic pathways with minimal toxicity. We now emphasize that existing strategies often struggle with balancing selectivity and potency across multiple targets, leading to suboptimal therapeutic outcomes. Our study addresses this gap by focusing on the rational design of [(2-Phenylindol-3-yl)methylene]propanedinitrile and 2-phenylindole-3-carbaldehyde derivatives, which offer favorable structural features such as a rigid indole core and functionalizable side chains that can be tailored for simultaneous interactions with CDK2, EGFR, and Tubulin.
Moreover, we expanded the rationale for selecting these specific derivatives, explaining that their planar aromatic system and versatile binding capabilities make them ideal scaffolds for multitarget design. We have also elaborated on the advantages of targeting CDK2, EGFR, and Tubulin simultaneously, noting that these proteins are key drivers of proliferation, survival, and metastasis in cancer. By inhibiting them concurrently, there is potential to achieve synergistic therapeutic effects, overcome resistance mechanisms, and reduce the likelihood of tumor escape pathways—an approach supported by recent multitarget inhibitor studies
Materials and Method
I noticed several critical areas that require more detail for reproducibility. For instance, the molecular docking and dynamics protocols lack key parameters such as grid dimensions, solvent conditions, and scoring functions used during docking. Similarly, the rationale for selecting specific parameters (e.g., the 1.0 nm box size or 100 ns simulation time) is missing. Including this information would make the study more transparent and allow others to replicate the work.
I also feel that the description of the CoMSIA modeling could be expanded. While the statistical metrics are mentioned, there is no discussion of how they compare to similar studies or why the SEHDA model was ultimately chosen. Moreover, the dataset preparation and validation steps for the QSAR models could be more detailed, particularly the choice of training and test sets and any external validation strategies used.
Thank you for your valuable feedback regarding the level of detail needed for reproducibility.
We have carefully revised the Materials and Methods section to include the missing details. Specifically, we have now provided the molecular docking parameters, including the grid box dimensions, center coordinates, solvent model used, and the scoring functions applied during docking. For molecular dynamics simulations, we have specified the type of solvent model (e.g., TIP3P water model), ion concentration, temperature and pressure coupling methods, and have justified the selection of the 1.0 nm box size and the 100 ns simulation time based on standard practices for achieving equilibrium and sufficient sampling of protein-ligand interactions.
Regarding the CoMSIA modeling, we have expanded the discussion to compare our statistical metrics (e.g., Q2, R2, R2pred​) with values reported in similar studies, demonstrating that our model performs competitively. Additionally, we clarified the rationale behind selecting the SEHDA model over others, emphasizing its superior predictive ability and balanced statistical parameters.
For the QSAR dataset preparation and validation, we have added a detailed description of the division of compounds into training and test sets, specifying the method used (e.g., random or rational splitting based on activity range and chemical diversity). We also outlined the external validation procedures employed, including the use of predictive R2 and applicability domain analysis to ensure the robustness and generalizability of the model.
Results and Discussion
The quality of the figures, particularly Figures 1 through 3, is notably poor, which significantly hinders the clarity and interpretation of the results. I strongly recommend that the authors replace all low-resolution figures with highresolution, clear images that effectively convey the information. Improving the quality of the figures will not only enhance readability but also ensure that the results are presented in a professional and comprehensible manner, meeting the standards expected for publication.
The presentation of the results could benefit from a more integrated discussion that links findings across the different methodologies. For instance, the CoMSIA results identify key structural features contributing to biological activity, but the narrative does not adequately connect these features to the docking and MD simulation outcomes. Establishing this link would provide a more cohesive understanding of how these compounds achieve their multitarget efficacy and stability. While the emphasis on T09 and T10 is justified by their superior performance, the discussion does not adequately explore the broader implications of multitarget inhibition. For example, the potential for synergistic effects across CDK2, EGFR, and Tubulin pathways or challenges such as off-target effects and drug resistance is not thoroughly addressed. Moreover, the results section could highlight how these findings compare to existing inhibitors or similar studies, which would contextualize the significance of the compounds’ performance. Including such comparisons and discussing the translational potential of T09 and T10, alongside their possible limitations, would enrich the narrative and provide a more balanced perspective.
Thank you very much for your thorough and constructive comments.
In response, we have replaced Figures 1 through 3 with high-resolution, clear images to ensure better readability and a more professional presentation of the results.
Additionally, we have revised the Results and Discussion sections to provide a more integrated narrative that connects findings across the different methodologies. Specifically, we now clearly link the key structural features identified by CoMSIA modeling to the molecular interactions observed in the docking and molecular dynamics simulation studies. This integrated approach offers a cohesive explanation of how the structural characteristics of the compounds contribute to their multitarget efficacy and stability.
We have also expanded the discussion to address the broader implications of multitarget inhibition, highlighting the potential for synergistic therapeutic effects across the CDK2, EGFR, and Tubulin pathways. Furthermore, we now discuss possible challenges such as off-target effects and drug resistance that may arise with multitarget strategies.
Conclusion
I feel that the claims about the compounds’ potential for clinical development are slightly overstated, as they are based solely on computational data. Including a statement about the need for experimental validation would provide a more balanced perspective and align the conclusion with the study’s scope.
Additionally, while the conclusion emphasizes the promising nature of T09 and T10, it does not clearly articulate what sets this study apart from previous research. Highlighting any unique insights or novel contributions to the field would strengthen the impact of the conclusion and provide a compelling case for publication.
Thank you for your thoughtful and important feedback.
In response, we have revised the conclusion to adopt a more balanced tone regarding the clinical potential of the compounds. We now clearly acknowledge that the findings are based solely on computational analyses and emphasize the necessity of experimental validation to confirm the predicted activities and multitarget efficacy of Pred9 and Pred10.
Furthermore, we have strengthened the conclusion by explicitly highlighting the unique contributions of our study. In particular, we emphasize the rational design of indole-based multitarget inhibitors specifically tailored to interact with CDK2, EGFR, and Tubulin, supported by integrated QSAR, docking, and molecular dynamics approaches. This integrated computational strategy and the identification of key structural features that simultaneously promote multitarget binding set our work apart from previous studies.
Reviewer #4:
The authors effectively examined the inhibitory potential of 2-phenylindol-3-yl)methylene] propanedinitrile and 2-phenylindole-3- carbaldehyde derivatives against three cancer-associated targets. However, I suggest that the authors address the following concerns.
Thank you for your valuable comment.
In response, we would like to clarify that the inhibitory activities of the synthesized compounds were collected against the MCF-7 breast cancer cell line. MCF-7 is a well-established model for studying breast cancer due to its expression of hormone receptors and relevance to human disease. This choice provided a reliable biological context to evaluate the antiproliferative potential of our derivatives.
1a. To make the introduction more robust and informative, the authors should include the types of cancer in which these targets are expressed and elaborate more on why targeting these targets is essential.
Thank you for your insightful comment.
We selected CDK2, EGFR, and Tubulin as targets due to their critical and complementary roles in breast cancer progression and therapy resistance. Specifically:
CDK2 regulates the G1/S transition of the cell cycle, and its overexpression in breast cancer leads to uncontrolled proliferation and resistance to therapy.
EGFR promotes tumor growth, survival, angiogenesis, and metastasis, particularly in aggressive subtypes such as triple-negative breast cancer (TNBC).
Tubulin is essential for mitosis, and disrupting its dynamics is a validated strategy to inhibit cancer cell division and induce apoptosis.
By simultaneously targeting these proteins, our approach aims to achieve synergistic therapeutic effects, overcoming the limitations of single-target therapies and potentially minimizing resistance.
In response to your suggestion, we have revised the Introduction accordingly to clearly articulate the rationale for choosing these targets and to better highlight their significance in the context of breast cancer treatment.
MOLECULAR DOCKING STUDIES.
2a. Section 2.5, The authors mentioned that “docking analysis highlights the efficacy of the proposed compounds relative to both the most active compounds in the dataset and FDA-approved reference drugs.” I suggest the authors include the reference drugs, briefly describe their limitations, and state the basis for investigating the inhibitory potential of 2-phenylindol-3-yl) methylene] propanedinitrile and 2-phenylindole-3- carbaldehyde derivatives.
Thank you for your thoughtful feedback. In the revised manuscript, we have addressed this comment in Section 2.5 (Introduction and Molecular Docking Studies) as follows:
We have included the names of the FDA-approved reference drugs used in the docking analysis: Roscovitine (for CDK2), Colchicine (for Tubulin), and Erlotinib (for EGFR).
Roscovitine: Although a known CDK2 inhibitor, it has demonstrated limited selectivity and modest clinical efficacy, primarily due to off-target effects.
Colchicine: Despite its effectiveness as a Tubulin inhibitor, its clinical use is restricted by significant toxicity and a narrow therapeutic index.
Erlotinib: A widely used EGFR inhibitor, but its therapeutic success is often limited by acquired resistance, including EGFR mutations and activation of alternative signaling pathways.
We have also stated the rationale for investigating the inhibitory potential of [(2-Phenylindol-3-yl)methylene]propanedinitrile and 2-phenylindole-3-carbaldehyde derivatives, emphasizing their potential to overcome the limitations of existing inhibitors through:
Multitarget activity, which may reduce the likelihood of resistance by simultaneously disrupting the CDK2, EGFR, and Tubulin pathways.Additionally, we clarified that the goal of this work is to improve the therapeutic profile of existing molecules by using the most active compound in the dataset, compound 5n, as a starting point for the design of more potent multitarget inhibitors.


Results and discussion
I suggest that the authors should arrange the tables accordingly. I observed that the authors started the table numbering from Table 1, Table 2, followed by Table 1 and Table 2 (which ought to be Tables 3 and 4 accordingly).  
Thank you for highlighting this oversight. In the revised manuscript, the tables have been carefully reviewed and renumbered sequentially to ensure proper organization and consistency accordingly
In Table 6, the authors discussed the docking score of the identified compounds against the three targets; I suggest that the authors include the names of the reference drugs instead of Ref (CDK2), Ref (tubulin), and Ref (EGFR).
Thank you for your valuable suggestion. In the revised manuscript, Table 7 has been updated to include the names of the reference drugs instead of the generic terms Ref (CDK2), Ref (Tubulin), and Ref (EGFR).
I noticed that sections 3.5 and 2.6 repeatedly explained molecular dynamics.
Thank you for your observation. In the revised manuscript, the redundancy between Sections 3.5 and 2.6 regarding molecular dynamics has been fully addressed.
I suggest the authors include future directions and run a grammatical check on the manuscript.
Thank you for your helpful suggestion. In response, we have revised the Conclusion section to include a discussion on future directions, outlining potential areas for further research. Additionally, we have thoroughly run a grammatical check on the manuscript to ensure clarity and improve readability.
